# Supplementary material for: High-Performance Surface-Enhanced Raman Scattering Substrates Based on the ZnO/Ag Core-Satellite Nanostructures
Source: Nanomaterials (Basel). 2022 Apr 10;12(8):1286. doi: 10.3390/nano12081286 (PMC9027200; doi:10.3390/nano12081286)
Supplement: Supplementary file 1 [file nanomaterials-12-01286-s001.zip › nanomaterials-1640186-supplementary.pdf]

Supplementary Materials

High-performance surface-enhanced Raman scattering substrates based on the ZnO/Ag core-satellite nanostructures

Qianqian Sun[a,1*], Yujie Xu[a,1], Zhicheng Gao[a], Hang Zhou[a], Qian Zhang[a], Ruichong Xu[a], Chao Zhang[1], Haizi Yao[b*] and Mei Liu[a*]

[1]Collaborative Innovation Center of Light Manipulations and Applications in Universities of Shandong, School of Physics and Electronics, Shandong Normal University, Jinan 250014, China; 2019020497@stu.sdnu.edu.cn (Y.X.); 201809020304@stu.sdnu.edu.cn (Z.G.); 2020020573@stu.sdnu.edu.cn (H.Z.); 202009100522@stu.sdnu.edu.cn (Q.Z.); 202009100431@stu.sdnu.edu.cn (R.X.)

[2] Key Laboratory of Smart Lighting in Henan Province, School of Energy Engineering, Huanghuai University, Zhumadian 463000, China

* Correspondence: qianqiansun@sdnu.edu.cn (Q.S.); yaohaizi@huanghuai.edu.cn (H.Y.); liumei@sdnu.edu.cn (M.L.)

† These authors contributed equally to this paper.

1. Experimental section

1.1. Materials

Zinc acetate dehydrate ($Zn(OAc)_2 \cdot 2H_2O$, ≥99.0%) and diethylene glycol (DEG,≥99.0%) were purchased from Sinopharm Chemical Reagent Co. Ltd.. Methanol (99.9%) was purchased from Alfa Aesr.

1.2. Preparation of ZnO@Ag nanospheres Raman substrates

Zinc oxide nanospheres (ZnO NSs) were synthesized by the pyrolysis method with a two-stage reaction process. In the first stage, the solution was typically prepared by dissolving $Zn(OAc)_2 \cdot 2H_2O$ (1.1 g) in DEG (50 mL) at 80 °C and then heated at 160 °C for 10 min under vigorous stirring. The precipitate (ZnO NSs) and supernatant (Zn-complex precursors) were separated by centrifuging the cooling solution. Polydisperse ZnO NSs (p-ZnO NSs) were obtained by washing with methanol twice at 1000 rpm. In the second stage, the same solution of $Zn(OAc)_2 \cdot 2H_2O$ (1.1 g) in DEG (50 mL) was prepared, and the additional supernatant (10 mL) was added into the solution immediately when it was heated up to 130 °C. The solution was further heated at 160 °C for 60 min. After centrifugation and washing process, the monodisperse ZnO NSs (m-ZnO NSs) were obtained. Then, the synthesized p-ZnO NSs and m-ZnO NSs were added to methanol solvent, respectively. The p-ZnO NSs methanol solution and m-ZnO NSs methanol solution were dripped onto the cleaned silicon substrates. Then, Ag with 6 nm deposition thicknesses was deposited on the surface of ZnO NSs by thermal evaporation under 10^{-4} Pa and the thickness was monitored by using a quartz crystal microbalance. Finally, a series of Raman substrates were obtained based on ZnO/Ag nanospheres.

1.3. Equipment and characterization

Scanning electron microscopy (SEM) images were obtained by using an SEM ZEISS Sigma 500 at 3 kV. The X-ray diffraction (XRD) pattern was recorded in Bruker D8 ADVANCE. The X-ray photoelectron spectroscopy (XPS) spectra were measured by using EscaLab 250Xi. The absorption spectra of the different structures on quartz glass were measured by using a PERSEE TU-1900 spectrophotometer with a wavelength range of 300–900 nm. The probe molecules R6G and MG were dissolved in ethanol to obtain the solution from 10^{-2} to 10^{-15} M, and from 10^{-3} to 10^{-13} M, respectively. Before the Raman test, the 2 μL of R6G, MG solution was dropped on the SERS substrate surface, and the ethanol can be completely evaporated. Raman spectra were

recorded by the Horiba HR Evolution 800 Raman microscope system using a 50× objective and laser of 532 nm with the power of 0.48 mW, and the integration time was 8 s throughout the experiment, where the diffraction grid was selected as 600 gr/mm.

2. Additional experimental results

Polydispersity index (Pdi) is used to describe the width of the particle size distribution. The Pdi would be related to the standard deviation (σ) of the hypothetical Gaussian distribution and the average size (Z_d). Pdi is Relative variance, which can be calculated by Pdi=$\frac{\sigma^2}{Z_d^2}$. The relative polydispersity can be calculated by % Polydispersity (%Pd) = Coefficient of variation = $(PDI)^{1/2}$ × 100. As a rule of thumb, samples with %Pd < 20% can be considered monodisperse. The σ of the diameter for p-ZnO NSs is 91 nm. The σ of the long diameter and the short diameter for the m-ZnO NSs are 27 and 20 nm, respectively. The σ for the diameter of p-ZnO NSs is 91 nm. The σ for the long diameter and the short diameter of m-ZnO NSs are 27 and 20 nm, respectively. The %Pd of diameter for p-ZnO NSs, long diameter, and short diameter for the m-ZnO NSs are 45.5%, 15.2%, and 16.7%, according to the average diameter of p-ZnO NSs (200 nm), the average long diameter (162 nm) and short diameter (132 nm) for the m-ZnO NSs, respectively.

The value of N_{RS}/N_{SERS} was estimated from the ratio of the average areal density (AD) of the R6G on the flat Si substrate and the fabricated Si/p-ZnO NSs/Ag NPs or Si/m-ZnO NSs/Ag NPs substrates due to the same laser spot of 1 μm. The calculation method of the average AD is as follows. The probe molecule R6G was dissolved in ethanol to obtain the solution from 10^{-2} to 10^{-15} M. The 2 μL R6G with the concentration 10^{-2} and 10^{-15} M were dropped separately on the flat Si substrate and the fabricated SERS substrates. Before carrying out the Raman detection, the ethanol of the R6G solution was completely evaporated. When the concentrations of R6G are lower than 10^{-14} M, the SERS signal of R6G can be only obtained from the edge of the final evaporation imprint. Thus, to guarantee the scientific nature of the results, the 10^{-13} M was chosen as the limit concentration of the R6G solution for the calculation of the enhancement factor (EF). The estimated maximum diameter of the final evaporation imprint on the flat Si substrate was around 6 mm for R6G (10^{-4} M). Additionally, the estimated maximum diameter of the final evaporation imprint was around 4 mm and 5 mm for R6G (10^{-13} M) for the fabricated Si/p-ZnO NSs/Ag NPs and Si/m-ZnO NSs/Ag NPs substrates, respectively. Thus, the AD of the R6G can be estimated by the following equation: AD =CVN/S, where C, V, N, and S represent the concentration of probe molecule solution, the volume of the probe molecule solution, Avogadro constant, and the area of the probe molecule, respectively. The AD of the R6G with a concentration of 10^{-4} M on the flat Si substrate is around 2.0×10^{7} molecules/μm^2. The ADs of the R6G with the concentration of 10^{-13} M on the fabricated Si/p-ZnO NSs/Ag NPs and Si/m-ZnO NSs/Ag NPs substrates are around 3.0×10^{-2} and 2.4×10^{-2} molecules/μm^2, respectively. The calculated EF of the Si/p-ZnO NSs/Ag NPs substrates is 2.6×10^{8} according to the $I_{SERS}/I_{RS}\approx0.40$, $N_{RS}/N_{SERS}\approx6.6\times10^{8}$. The calculated EF of the Si/m-ZnO NSs/Ag NPs substrates is 2.5×10^{8} according to the $I_{SERS}/I_{RS}\approx0.30$, $N_{RS}/N_{SERS}\approx8.3\times10^{8}$.

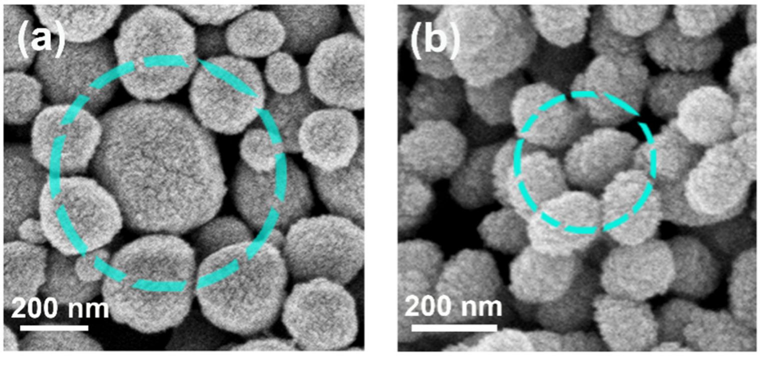

Figure S1. Magnified SEM images of p-ZnO NSs (**a**) and m-ZnO NSs (**b**).

Table S1. Vibrational mode for characteristic peaks of R6G.

Raman shifts (cm^{-1})	Vibrational mode
**613**	C-C-C ring in-plane bending mode
**774**	C-H out-of-plane bending mode
**1185**	C-H in-plane bending mode
**1365**	aromatic C-C stretching mode
**1508**	aromatic C-C stretching mode
**1650**	aromatic C-C stretching mode

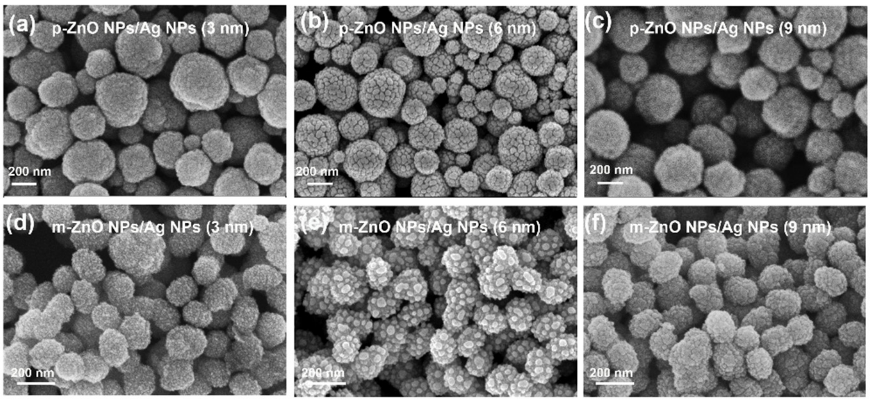

Figure S2. SEM images of p-ZnO NSs/Ag NPs (**a**-**c**) and m-ZnO NSs/Ag NPs (**d**-**f**) with different deposition thicknesses (3 nm, 6 nm, 9 nm) of Ag by thermal evaporation.

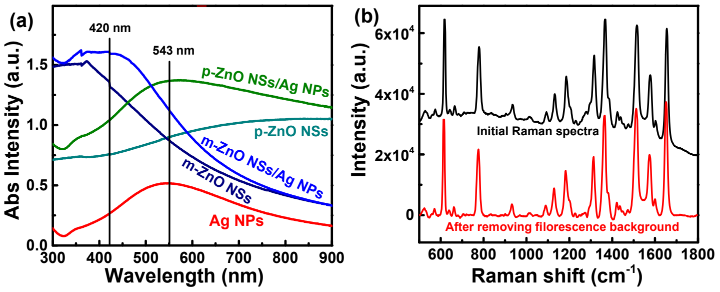

Figure S3. (**a**) Absorption spectra of the different structures on quartz glass. (**b**) Initial Raman spectra and Raman spectra after removing fluorescence background for R6G with a concentration of 10^{-7} M on the Si/p-ZnO NSs/Ag NPs substrates.

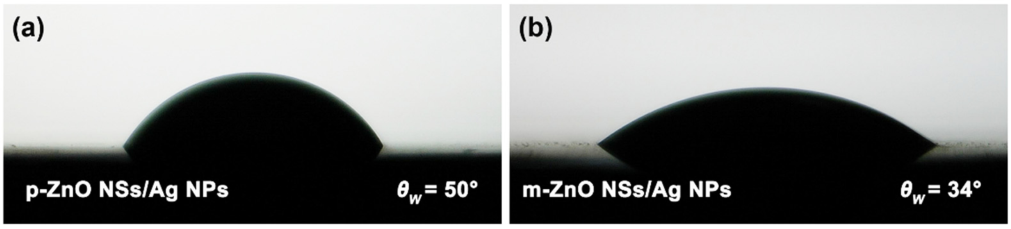

Figure S4. Contact angle images of Si/p-ZnO NSs/Ag NPs and Si/m-ZnO NSs/Ag NPs substrates with water as the probing liquid.

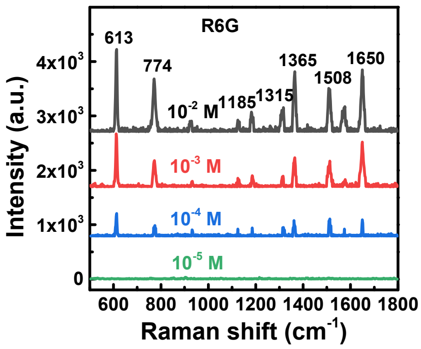

Figure S5. Raman spectra of R6G with concentrations of 10^{-2} ~ 10^{-5} M on the flat Si substrates.

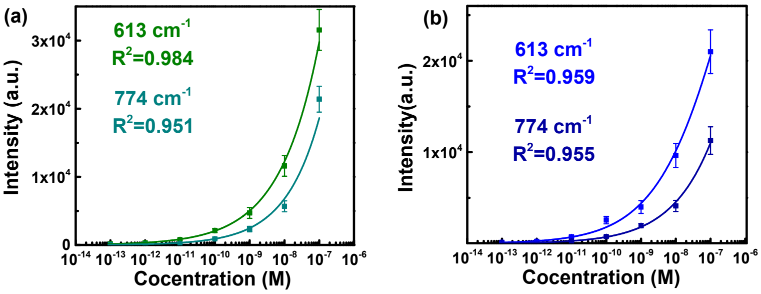

Figure S6. Non-linear Langmuir isotherm fitting curve between Raman intensities of 613 cm^{-1} peaks and the logarithm of the concentrations of R6G (10^{-7}~10^{-13} M) for Si/p-ZnO NS/Ag NP (**a**) Si/m-ZnO NS/Ag NP (**b**) substrates.

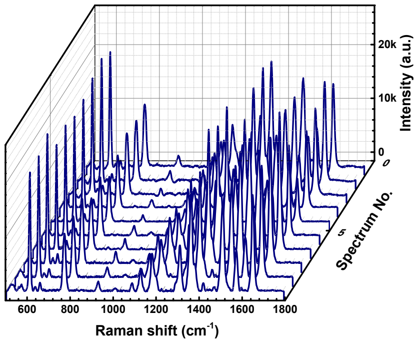

Figure S7. Ten Raman spectra of R6G with a concentration of 10^{-7} M on the Si/m-ZnO NS/Ag NP substrate from different batches.

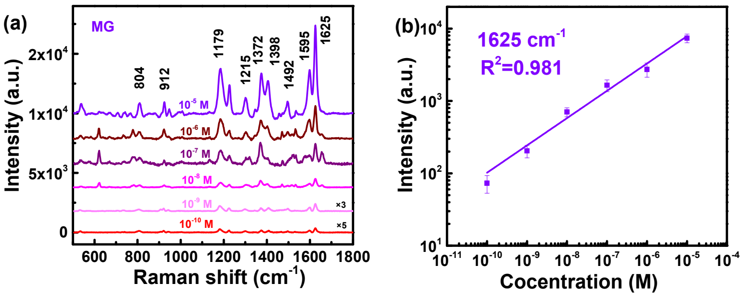

Figure S8. (**a**) Raman spectra of MG with different concentrations on the Si/p-ZnO NS/Ag NP substrates. (**b**) Calibration curve of Raman intensity at 1645 cm^{-1} versus the concentration of MG.
